# Supplementary material for: SINE Insertion May Act as a Repressor to Affect the Expression of Pig LEPROT and Growth Traits
Source: Genes (Basel). 2022 Aug 10;13(8):1422. doi: 10.3390/genes13081422 (PMC9407865; doi:10.3390/genes13081422)
Supplement: Supplementary file 1 [file genes-13-01422-s001.zip › genes-1831450-supplementary.pdf]

Table S1 Primers used for identification of RIPs, expression and vector construction

Primer name	Primer sequence (5′-3′)	Size	Annealing temperature	Purpose
LEPROT-SINE- RIP	F: GGGGTACCCGCACCTTGGATCCCACA<br>R: CGGCTAGCTTCCCTGCATCTACCGGACA	460bp	60℃	RIPs
LEPR-SINE- RIP1	F: CTGTTCAGTCCCTAGTGCTT<br>R: TCTTATGTAGATGTGTCACGG	865bp	55℃	identification
LEPR-SINE- RIP2	F: AAAGTGAAAGTTTTAGCCAACC<br>R: GGCATCCTTGTCTTGTTCT	673bp	55℃	
GAPDH	F: GGACTCATGACCACGGTCCAT<br>R: TCAGATCCACAACCGACACGT	220bp	58℃	Expression
LEPROT	F: AGCTCTTGTGGCTTTATCCTT<br>R: CCACACGAGCAAGAATAACGG	260bp	58℃	
LEPROT$^{SINE+/-}$-Luc^{+}(EN)	F: GgggtaccccCTACCTTTAGCAAAGAGCGTT<br>R: CggctagccgCCTGCATCTACCGGACACT	878/602bp	60℃	Vector construction

Table S2 Number and origin of pig breeds for RIPs identification and distribution

Breed	Number	Province/country of origin
Duroc	24	
Landrace	24	Anhui/China
Large white	450	
Sujiang	24	Jiangsu/China
Jiangquhai	24	Jiangsu/China
Jinhua	24	Zhejiang/China
Rongchang	24	Chongqing/China
Erhualian	24	Jiangsu/China
Bama	24	Guangxi/China
Wuzhishan	24	Hainan/China
Diannan small-ear	6	Yunnan/China
Tibetan	6	Sichuan/China
Meishan	6	Jiangsu/China
Fengjing	6	Jiangsu/China
Wild boars	12	Fujian/ Heilongjiang/Anhui /China

Table S3 Predicted structural variations of the porcine *LEPTIN*、*LEPROT* and *LEPR* genes using Clustal X and RepeatMasker

	LEPTIN	*LEPROT*	*LEPR*	Total

SVs	8	10	36	54
SINE	3	5	15	23
LINE	0	1	4	5
LTR	0	0	4	4
RIPs/SVs	37.50%	60.00%	63.89%	59.26%

Note: the sequences of structural variation in this experiment were greater than or equal to 50 bp.
